# Cross-Talk Between m^6^A- and m^5^C-Related lncRNAs to Construct a Novel Signature and Predict the Immune Landscape of Colorectal Cancer Patients

**DOI:** 10.3389/fimmu.2022.740960

**Published:** 2022-03-08

**Authors:** Wei Song, Jun Ren, Rensheng Xiang, Wenzheng Yuan, Tao Fu

**Affiliations:** Department of Gastrointestinal Surgery II, Renmin Hospital of Wuhan University, Wuhan, China

**Keywords:** colorectal cancer, long non-coding RNA, m^6^A regulators, m^5^C regulators, tumor microenvironment, immunotherapy

## Abstract

**Background:**

N6-methyladenosine (m^6^A) and 5-methylcytosine (m^5^C) can modify long non-coding RNAs (lncRNAs), thereby affecting tumorigenesis and tumor progression. However, there is a lack of knowledge regarding the potential roles and cross-talk of m^6^A- and m^5^C-related lncRNAs in the tumor microenvironment (TME) and their effect on prognosis.

**Methods:**

We systematically evaluated the expression patterns of m^6^A- and m^5^C-related lncRNAs in 1358 colorectal cancer (CRC) samples from four datasets. Consensus clustering was conducted to identify molecular subtypes of CRC, and the clinical significance, TME, tumor-infiltrating immune cells (TIICs), and immune checkpoints in the different molecular subtypes were analyzed. Finally, we established a m^6^A- and m^5^C-related lncRNA signature and a prognostic nomogram.

**Results:**

We identified 141 m^6^A- and m^5^C-related lncRNAs by co-expression analysis, among which 23 lncRNAs were significantly associated with the overall survival (OS) of CRC patients. Two distinct molecular subtypes (cluster A and cluster B) were identified, and these two distinct molecular subtypes could predict clinicopathological features, prognosis, TME stromal activity, TIICs, immune checkpoints. Next, a m^6^A- and m^5^C-related lncRNA signature for predicting OS was constructed, and its predictive capability in CRC patients was validated. We then constructed a highly accurate nomogram for improving the clinical applicability of the signature. Analyses of clinicopathological features, prognosis, TIICs, cancer stem cell (CSC), and drug response revealed significant differences between two risk groups. In addition, we found that patients with a low-risk score exhibited enhanced response to anti-PD-1/L1 immunotherapy. Functional enrichment analysis showed that these lncRNAs related to the high-risk group were involved in the development and progression of CRC.

**Conclusions:**

We conducted a comprehensive analysis of m^6^A- and m^5^C-related lncRNAs in CRC and revealed their potential functions in predicting tumor-immune-stromal microenvironment, clinicopathological features, and prognosis, and determined their role in immunotherapy. These findings may improve our understanding of the cross-talk between m^6^A- and m^5^C-related lncRNAs in CRC and pave a new road for prognosis assessment and more effective immunotherapy strategies.

## Introduction

Colorectal cancer (CRC) is one of the most common and lethal cancers of the digestive system, and it remains a challenging issue globally (1). Colonoscopy is available currently, and such early screening methods can effectively prevent the occurrence of CRC, but its hidden onset, long evolution time, and high malignancy grade have frequently led to poor prognosis (2, 3). CRC is characterized by inherent biological invasiveness as well as specific radiological and chemical resistance that result in high recurrence rates and progression in patients. Although different treatments, such as surgery, chemotherapy, radiotherapy, and some new immunotherapies, are currently applied, their clinical benefits remain unsatisfactory (4). Therefore, efficient prognostic biomarkers and functional signatures may be beneficial to realize individualized survival predictions and provide patients with an optimal therapeutic approach.

Long non-coding RNAs (lncRNAs), a sequence made up of more than 200 bp but lacking protein encoding capability, are transcribed by RNA polymerase II; they play a crucial regulatory role at the transcriptional, post-transcriptional, and epigenetic levels, and are involved in multiple aspects of gene regulation and numerous biological processes (5). Accumulating evidence has shown that lncRNAs can directly combine with DNA, RNA, or proteins to regulate gene expression in the form of RNA at various levels, thus resulting in the alteration of multiple physiological and pathological processes, including cell proliferation, migration, metabolism, and immunity (6, 7). Many researchers have explored the lncRNA expression profile of CRC and found that lncRNAs can serve as biomarkers of CRC prognosis and diagnosis (8, 9). Of note, lncRNA modification can change transcript stability and gene expression, causing regulatory abnormalities, which in turn influence tumorigenesis and cancer progression (10).

To date, more than 170 post-transcriptional modifications in RNA have been discovered, most of which are distributed in highly abundant non-coding RNAs (ncRNAs), such as transfer RNA (tRNA) and ribosomal RNA (rRNA), and small nuclear RNA (snRNA), and are involved in ncRNA biogenesis, metabolism, and other biological functions (11, 12). Among these modifications, the most frequent are N6-methyladenosine (m^6^A), 5-methylcytosine (m^5^C), N1-methyladenosine (m^1^A), adenosine to inosine transition (A-to-I), and pseudouridine (Ψ). To date, several chemically modified lncRNAs have been identified in cancers (12), and there may be some competitive compensation interactions between these modifications.

m^6^A modification, the most common post-transcriptional modification of mRNAs and ncRNAs, plays a vital role in RNA maturation, export, stability, translation, export, and decay (13, 14). To date, m^6^A modifications have been identified in more than 7,600 genes and 300 non-coding RNAs in mammals (10). As an invertible and dynamic RNA epigenetic process, the molecular components of m^6^A include intracellular methyltransferases (“writers”), demethylases (“erasers”), and signal transducers (“readers”), which regulate gene expression and are associated with various biological functions, such as RNA splicing, export, stability, translation, and ncRNA biogenesis (15, 16). Accumulating evidence has shown that changes in m^6^A modification patterns are related to the tumorigenesis and progression of various types of cancer (17–19). Abnormal m^6^A methylation levels can affect the self-renewal of cancer stem cells, tumor immune response, microRNA (miRNA) editing, promotion of cancer cell proliferation, and resistance to radiotherapy or chemotherapy (20–23). For example, mediated by m^6^A modification, CBX8 interacts with KMT2b and Pol II to promote LGR5 expression, which contributes to increasing cancer stemness and decreasing chemosensitivity in colon cancer (22). METTL3-mediated m^6^A modification was found to promote the proliferation of bladder cancer by promoting pri-miR221 and pri-miR222 maturation (23). YTHDF3 recognizes and binds to m^6^A-modified lncRNA GAS5, promoting its degradation, which elevates YAP expression and exacerbates CRC (24).

m^5^C is another abundant RNA modification in humans (25). It occurs when the fifth carbon of RNA cytosine is modified by methylation (26). This modification was first reported in rRNA, and later reported in other RNAs, such as mRNAs and ncRNAs (rRNAs, tRNAs, lncRNAs, and eRNAs) (27). The distribution of m^5^C methylation modification varies greatly in different kinds of RNAs and species. Like other type of RNA methylation, m^5^C methylation modification is a reversible process regulated by methylases and demethylases, and can only be biologically activated by methylation binding proteins (26). Just like m^6^A, m^5^C plays critical roles in RNA stability, translation, and nuclear transport (25, 28). Aberrant expression of m^5^C has been found to play carcinogenic roles in several cancers, including gastric cancer (29), bladder cancer (28), and pancreatic cancer (30).

The tumor microenvironment (TME) has been recognized as an important component of malignant tumor tissues and plays various roles in tumorigenesis, tumor progression, metastasis, treatment resistance, and disease recurrence (31). The complex interaction between tumor cells and the TME plays an essential role in tumor development. Tumors can affect their microenvironment, promoting tumor angiogenesis and inducing immune tolerance by releasing cell signaling molecules. The TME can regulate cancer progression, and tumor-infiltrating immune cells (TIICs) within this environment are reported to be of great value in predicting cancer prognosis (32).

To fully elucidate how the regulatory network of m^6^A- and m^5^C-related lncRNAs affects the TME, there is an urgent need for understanding the crosstalk between these different patterns of changes in lncRNAs. The two RNA modifications in lncRNAs may form an important and complex cellular regulatory network in CRC. The understanding of this network may provide important insights into the underlying mechanism of CRC tumorigenesis and may open up new therapeutic possibilities for CRC. In the present study, we explored genomic alterations in 1358 CRC samples from The Cancer Genome Atlas (TCGA) and Gene Expression Omnibus (GEO) datasets to comprehensively evaluate the roles of m^6^A- and m^5^C-related lncRNAs. We revealed two distinct molecular subtypes that can be used to predict clinicopathological features, prognosis, TME stromal activity, TIICs, and immune checkpoints. We further established a set of scoring system to predict OS for CRC patients. The current investigation will contribute to great progresses on the exploration of prognostic m^6^A- and m^5^C-related lncRNAs and shed new light on the possible mechanisms of CRC development.

## Materials and Methods

### Data Sources

**Figure 1:**
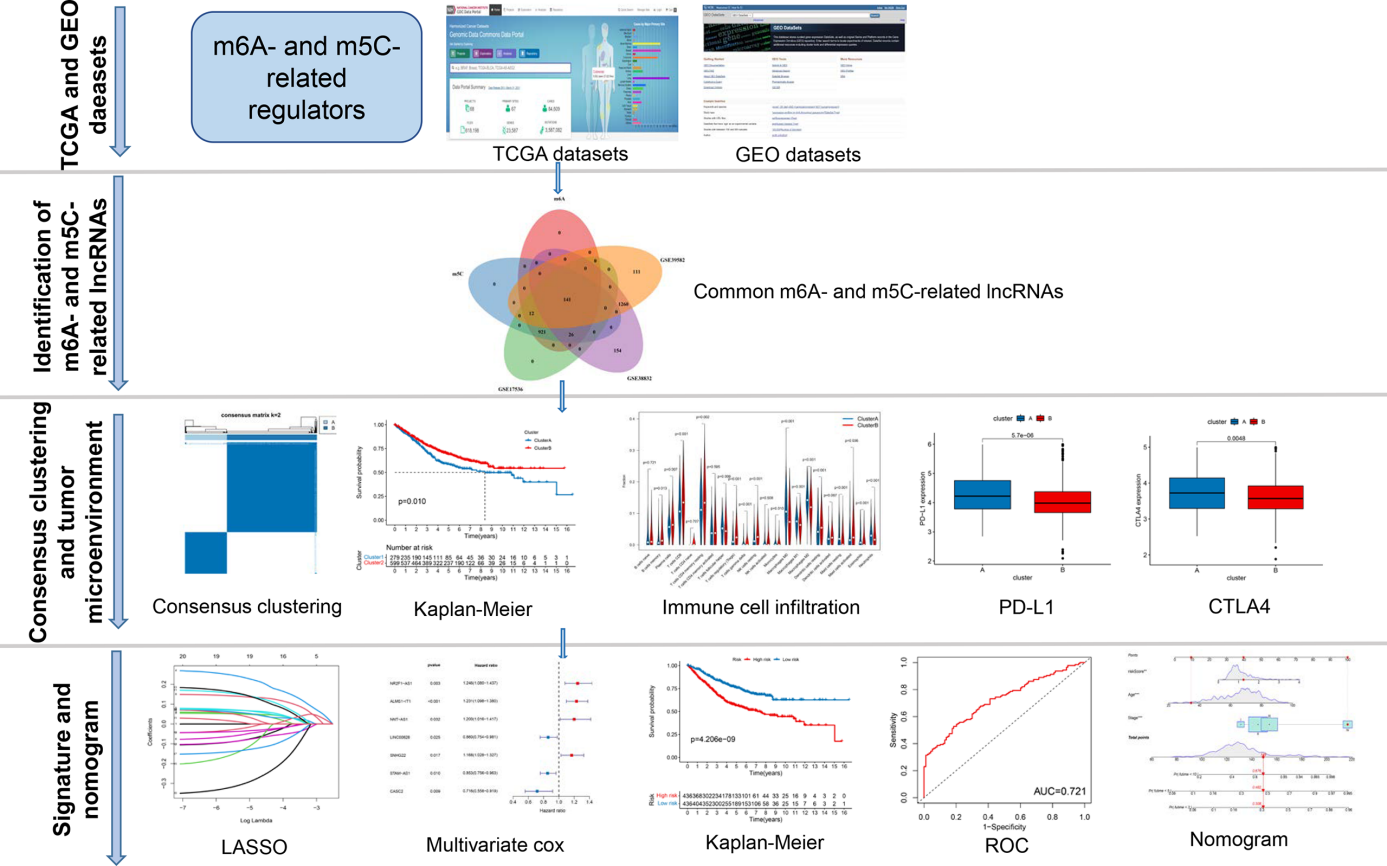
The entire analytical process of the study.

A map illustrating the process of this study is shown in **Figure 1**. Gene expression (fragments per kilobase million, FPKM) and the relevant the corresponding prognostic and clinicopathological data of CRC were downloaded from The Cancer Genome Atlas (TCGA) database (https://portal.gdc.cancer.gov/) and the gene expression omnibus (GEO) database (https://www.ncbi.nlm.nih.gov/geo/). GTF files were downloaded from Ensembl (http://asia.ensembl.org/index.html) for accurate distinguishing of mRNAs and lncRNAs. Three GEO CRC cohorts (GSE39582, GSE17536, GSE38832) and TCGA cohort were obtained for subsequent analysis (**Table S1**). We downloaded the raw “CELL” files and performed background adjustment and quantile normalization. The FPKM values of TCGA-COAD/READ were transformed into Transcripts Per kilobase Million (TPM) as previously described. Three GEO datasets were combined, and the “Combat” algorithm was used to eliminate the batch effect. We excluded data from patients who had no survival information. Detailed information on CRC patients is shown in **Table S1**. Clinical variables involved age, sex, tumor location, TNM stage, KRAS mutation, BRAF mutation, follow-up time, and survival status.

### Identification of m^6^A- and m^5^C-Related lncRNAs

Based on published data, 23 recognized m^6^A and 15 m^5^C regulators were obtained, including writers, erasers, and readers. The list of m^6^A and 15 m^5^C regulators is provided in **Table S2**. The correlation between lncRNAs and the expression of m^6^A- and m^5^C-related genes was analyzed using the Pearson correlation test. LncRNAs with |correlation coefficients| > 0.4 and P < 0.001 were identified as m^6^A- and m^5^C-related lncRNAs, respectively. Next, the intersection between the m^6^A- and m^5^C-related lncRNAs was considered as a candidate lncRNA. To screen for m^6^A- and m^5^C-related lncRNAs that were highly correlated with OS, univariate Cox regression analysis was performed (P < 0.05) based on lncRNAs from three GEO datasets.

### Consensus Clustering Analysis of m^6^A- and m^5^C-Related lncRNAs

The “ConsensusClusterPlus” package in R was used for consensus unsupervised clustering analysis to classify patients into distinct molecular subtypes according to the expression of m^6^A- and m^5^C-related lncRNAs obtained from univariate Cox regression analysis. Clustering was conducted based on the following criteria. Firstly, the cumulative distribution function curve increased gradually and smoothly. Secondly, there was no group with a small sample size. Lastly, after clustering, the intra-group correlation increased, whereas the inter-group correlation decreased. To investigate the differences in biological processes between m^6^A- and m^5^C-related lncRNAs, we conducted Gene Set Variation Analysis (GSVA) enrichment analysis using the “GSVA” R package. The gene set “c2.cp.kegg.v7.2” and “clusterProfiler” R package was used to perform functional annotation for m^6^A- and m^5^C-related lncRNAs, with the cutoff value of adjusted p-value < 0.05.

### Clinical Significance of the Molecular Subtypes

To explore the clinical significance of the molecular subtypes in CRC, we investigated the relationship between the molecular subtypes, clinical characteristics, and prognosis. The patient characteristics included age, sex, tumor location, TNM stage, KRAS mutation, and BRAF mutation. Subsequently, the differences in OS between different clusters were calculated using the Kaplan-Meier method and visualized by using the “survival” and “survminer” modules in the R software.

### Evaluation of TME and TIICs

We employed the Estimation of Stromal and Immune cells in Malignant Tumors using Expression algorithm (ESTIMATE) to evaluate the immune score and stroma score of each CRC sample (33). In addition, the CIBERSORT (https://cibersort.stanford.edu/) algorithm was utilized to precisely measure the fractions of 22 human immune cell subsets in CRC samples (34).

### Establishment and Evaluation of the m^6^A- and m^5^C-Related lncRNA Signature

A total of 878 CRC patients from the three GEO datasets were used to construct a m^6^A- and m^5^C-related prognostic signature. Briefly, based on m^6^A- and m^5^C-related prognostic lncRNAs identified by univariate Cox regression analysis, the Lasso Cox regression algorithm was used to minimize the risk of over-fitting and remove highly related genes using the “glmnet” R package. A 10-fold cross validation was conducted to identify the optimal lambda value. Next, the screened lncRNAs were subjected to multivariate Cox proportional hazard regression analysis to obtain the optimal lncRNAs and establish a prognostic signature using the training set.

The following formula was used:


Risk Score=Σ(Expi∗Coefi)

*Expi* is the expression level of the lncRNA, and *Coefi* is the estimated regression coefficient of the lncRNA. The median value of the risk score was used as the cutoff for the risk score, and the patients were assigned to a high- (risk score > median value) or low-risk group (risk score > median value). We performed survival analysis between the two risk groups to detect whether the difference in OS was subsistent. A receiver operating characteristic (ROC) curve was also generated for further assessment of the predictive ability of the signature. Moreover, the accuracy of the model was validated using the TCGA cohort by the same method. To investigate whether the signature can predict patient response to immune checkpoint blockade therapy, the IMvigor210 cohort was downloaded from the website http://research-pub.gene.com/IMvigor210CoreBiologies/, which is a study cohort of atezolizumab in patients with locally advanced or metastatic urothelial carcinoma (35).

### Tissue Samples

A total of six pairs of colorectal tissue samples and paired adjacent normal colorectal tissues derived from surgically resected specimens were stored at –70°C until expression analysis. Tissues were attained during surgery prior to receiving chemo/radiotherapy. The study was approved by the ethical committee of the Renmin Hospital of University of Wuhan University. All subjects gave written informed consent in accordance with the Declaration of Helsinki.

### RNA Isolation and Quantitative Real Time-PCR (RT-qPCR)

Total RNA from tissues of CRC patients was extracted using the TRIzol reagent (Invitrogen, Carlsbad, CA, USA) according to the manufacturer’s instructions. We synthesized cDNA from RNA using the PrimeScript RT reagent kit (Takara, Japan). qPCR analysis was conducted with SYBR Green Premix Ex Taq (TaKaRa, Japan) and according to the standard program on CFX-96 (Bio-Rad Laboratories, Inc., USA). Gene expression levels were normalized to GAPDH levels, and the relative expression level was calculated using the 2^-ΔΔCq^ method.

### Clinical Correlation and Stratification Analyses of the Prognostic Signature

To explore the association of the signature with the clinicopathological features (age, sex, tumor location, TNM stage, KRAS mutation, and BRAF mutation) of CRC, the correlation between the signature and clinicopathological variables of CRC was assessed using the Chi-square test and visualized using the “pheatmap” package and “ggpubr” in the R software. To assess whether risk scores and clinicopathological characteristics can be used as independent prognostic factors, we subjected the training and testing sets to univariate and multivariate cox regression analyses. Moreover, stratified analysis was carried out to confirm whether the signature retains its predictive ability in various subgroups. These variables include age (< 60 and > 60 years), gender (female and male), tumor stage (I-II and III-IV), T stage (T1-2 and T3-4), N stage (N0 and N1-3), M stage (M0 and M1), tumor location (left-side and right-side), KRAS mutation (yes and no), and BRAF mutation (yes and no). To quantitatively evaluate CRC prognosis in clinical practice, a nomogram was generated by the “rms” package based on the outcome of the independent prognosis analysis. In the nomogram scoring system, each variable was matched with a score, and the total score was obtained by adding the scores across all variables of each sample. Time-dependent ROC curves for 3, 5, and 10 years were used to evaluate the nomogram. Calibration curves were drawn to depict the predictive value between the predicted 3-, 5-, and 10-year survival events and the virtual observed outcomes.

### Drug Sensitivity and Cancer Stem Cell (CSC) Analyses

To explore differences in therapeutic effects of chemotherapeutic drugs in patients across the high- and low-risk groups, R package “pRRophetic” was used to predict the half-maximal inhibitory concentration (IC50), which could construct a ridge regression signature based on TCGA gene expression profiles and Genomics of Drug Sensitivity in Cancer cell line expression spectrum (36). In addition, we also analyzed the relationship between the risk score and cancer stem cell (CSC).

### Functional Enrichment Analysis

To explore the differences in biological process between the different risk groups, we performed GO and KEGG pathway analysis using the “clusterProfiler” R package. Samples with p-value < 0.05 were considered significantly enriched.

### Statistical Analyses

All statistical analyses were implemented using R version 4.1.0. Statistical significance was set at p-value < 0.05. The Kruskal-Wallis test was used to analyze differences between three or more groups. The results of immune infiltration assay were analyzed using the ‘gsva’ package in R. The Kaplan-Meier plot was used to generate survival curves, and Log-rank test was performed to evaluate significant differences. Univariate and multivariate Cox proportional hazard regression analyses were utilized to determine whether the m^6^A- and m^5^C-related lncRNA signature can be an independent prognostic factor. For visual risk prediction, the nomogram was created by the “Survival” and “RMS” packages of R.

## Results

### Identification of m^6^A- and m^5^C-Related lncRNAs in CRC

**Figure 2:**
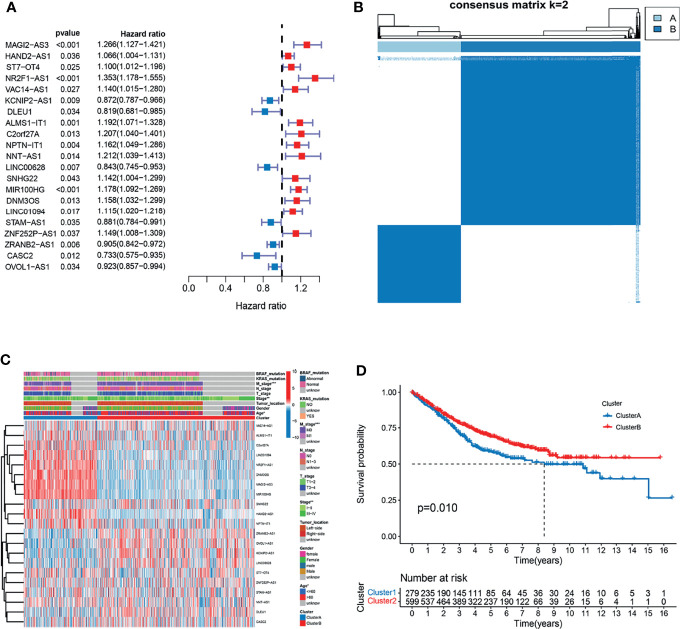
Analysis of the clinical characteristics, outcome, and expression level of m^6^A- and m^5^C-related lncRNAs between two distinct subtypes of samples divided by consistent clustering. **(A)** Univariate analysis revealed 21 lncRNAs related to overall survival (OS) time. **(B)** Consensus clustering analysis and the correlation area of the clusters when k = 2. **(C)** Differences in clinicopathologic features and the expression level of m^6^A- and m^5^C-related lncRNAs between the two distinct subtypes. **(D)** Kaplan-Meier curves of OS for patients with the two colorectal cancer (CRC) subtypes.

The entire analytical process used in this research is displayed in **Figure 1**. The gene expression and corresponding clinicopathological data were downloaded from the TCGA and GEO databases. To gain a comprehensive understanding of the expression patterns of the m^6^A- and m^5^C-related lncRNAs involved in tumorigenesis, 1364 CRC samples from four eligible CRC cohorts, namely TCGA-COAD/READ (n = 486), GSE39582 (n = 579), GSE17536 (n = 177), and GSE38832 (n = 122), were integrated in our study for further analysis. Detailed information on 1364 CRC patients is shown in **Table S1**. We extracted 14087 lncRNAs from the TCGA database in total, and 23 were recognized as m^6^A and 15 as m^5^C regulators from previous publications, respectively. Through co-expression analysis, we identified 1524 m^6^A- and 1581 m^5^C-related lncRNAs (|correlation coefficient| > 0.4, p-value < 0.001), respectively (**Table S3**). Consequently, a total of 1401 common lncRNAs were selected from the intersection of 1524 m^6^A- and 1581 m^5^C-related lncRNAs (**Figure S1A**). After intersecting lncRNAs obtained from three other GEO datasets, a total of 141 common lncRNAs of m^6^A- and m^5^C-related lncRNAs were identified and used for subsequent analysis (**Figure S1B**; **Table S4**). To confirm whether the 141 lncRNAs were correlated with the OS of CRC patients, we performed univariate Cox regression analysis based on the patients from three GEO datasets to identify the prognostic value of the m^6^A- and m^5^C-related lncRNAs. Twenty-one lncRNAs related to OS time (p-value < 0.05) were screened out and applied in the following analysis (**Figure 2A**).

### Consensus Clustering Analysis for m^6^A- and m^5^C-Related lncRNAs

To understand the effect of m^6^A- and m^5^C-related lncRNAs on CRC development, consensus unsupervised clustering analysis was conducted on the expression level of 21 m^6^A- and 15 m^5^C-related lncRNAs obtained from univariate Cox regression analysis. The optimal number of clusters was determined according to the cumulative distribution function and clinical significance. We choose the value of k = 2 as the appropriate number of clusters for further analysis. Finally, two subtypes were determined and dubbed cluster A (n = 279) and cluster B (n = 599) (**Figure 2B**).

### Correlation of Molecular Subtypes to Characteristics and Survival

To further examine the clinicopathological characteristics of the two subtypes identified by consensus clustering, the clinicopathological features of different subtypes of CRC patients were compared. Significant differences in the expression of m^6^A- and m^5^C-related lncRNAs and clinicopathological characteristics between the two subtypes were observed (**Figure 2C**). As shown in **Figure 2C**, Cluster B was preferentially related to younger age (p-value < 0.05) and lower TNM stage (p-value < 0.05), compared to cluster A. In addition, prognostic analysis of the two subtypes showed that patients with subtype B had longer OS than their counterparts with subtype A (**Figure 2D**). Taken together, clustering subtypes are significantly correlated with the heterogeneity of CRC.

### Characteristics of TME and TIICs in Distinct Subtypes

**Figure 3:**
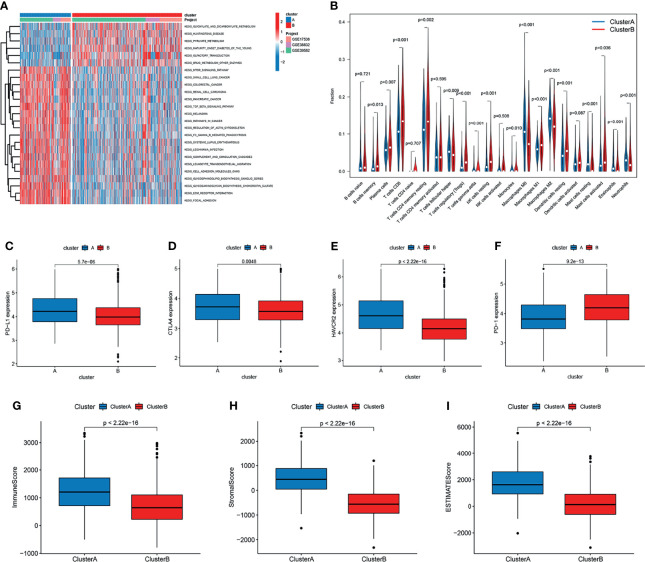
Correlation of tumor immune cell microenvironment to two colorectal cancer (CRC) subtypes. **(A)** GSVA enrichment analysis showing the activation states of biological pathways in the two distinct subtypes. The activated pathways are marked with red color, and the inhibited pathways are marked with blue color. **(B)** The infiltration levels of 22 immune cell types in the two subtypes. **(C–F)** The expression of tumor immune checkpoints (PD-L1, CTAL-4, HAVCR2, and PD-1), in the two subtypes. **(G–I)** TME score (stromal score, immune score, and estimate score) in the two subtypes.

To identify the biological significance of the two distinct subtypes, we conducted GSVA enrichment analysis. Cluster A was significantly enriched in stromal and oncogenic activation pathways, such as the ECM receptor interaction, colorectal cancer, TGF-β signaling pathway, cell adhesion, and other cancer-related pathways (**Figure 3A**). Cluster B was enriched in pathways related to metabolism, including glyoxylate and dicarboxylate metabolism, pyruvate metabolism, and Drug metabolism-other enzymes (**Figure 3A**).

To investigate the role of m^6^A- and m^5^C-related lncRNAs in the TME of CRC, we analyzed the relationship between the two subtypes and 22 human immune cell subsets of every CRC sample by the CIBERSORT algorithm (**Table S5**). The results revealed the differences in the infiltration level of most immune cells between the two subtypes (**Figure 3B**). Violin plots showed that follicular helper T cells, M0, M1, and M2 macrophages, resting mast cells, eosinophils, and neutrophils exhibited significantly higher infiltration rates in the subtype A group than in the subtype B group, whereas memory B cells, plasma cells, CD8+ T cells, resting memory CD4+ T cells, regulatory T cells, gamma delta T cells, resting NK cells, monocytes, resting dendritic cells, and activated mast cells had significantly lower infiltration rates in the subtype A group than in the subtype B group (**Figure 3B**). Similarly, the expression levels of PD-L1, CTAL-4, and HAVCR2 in cluster A were higher, and the expression levels of PD1 were lower (**Figures 3C–F**). In addition, we evaluated the TME score (stromal score, immune score, and estimate score) of the two subtypes by the ESTIMATE package. Higher stromal scores or immune scores represented higher relative content of stromal cells or immunocytes in the TME, and estimate scores indicated the aggregation of stromal scores or immune scores in the TME. The results showed that cluster A was significantly correlated to stromal score, immune score, and estimate score (**Figures 3G–I**).

### Construction of m^6^A- and m^5^C-Related lncRNA Prognostic Signature

**Figure 4:**
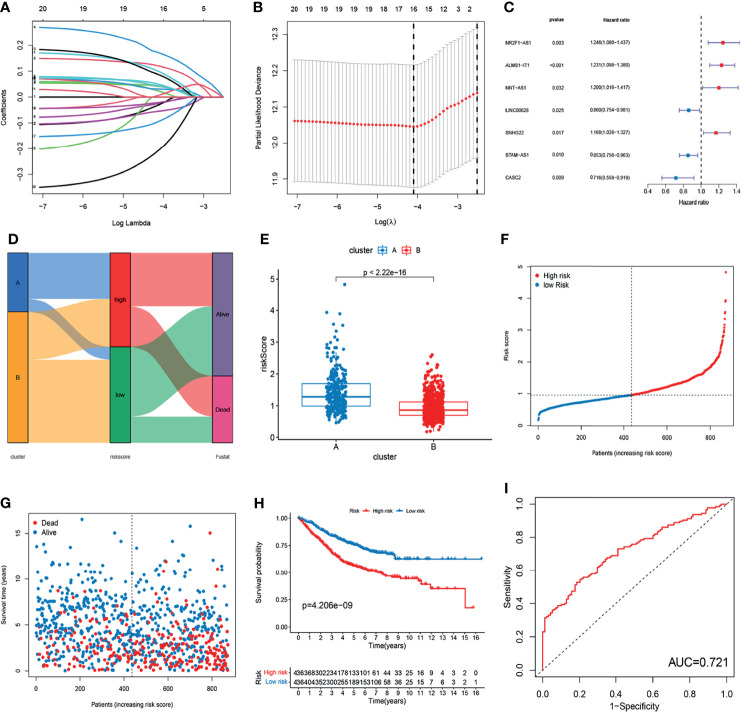
Construction of m^6^A- and m^5^C-related lncRNA signature using the training set. **(A, B)** LASSO regression analysis and partial likelihood deviance of prognostic lncRNAs. **(C)** Forest plot of multivariate cox regression analysis of prognostic lncRNAs. **(D)** Alluvial diagram of subtypes distribution in groups with different risk score and survival outcomes. **(E)** Correlation between the two subtypes and the different risk scores of the signature. **(F, G)** The ranked dot plot indicates the risk score distribution and scatter plot presenting patient survival status. **(H)** KM analysis of overall survival (OS) between the two groups. **(I)** Receiver operating characteristic (ROC) curve of the m^6^A- and m^5^C-related lncRNA signature.

To further explore the prognostic value of m^6^A- and m^5^C-related lncRNAs in CRC, LASSO Cox regression and multivariate Cox proportional hazard regression analyses for those 21 m^6^A- and m^5^C-related lncRNAs were conducted to further select a robust and effective risk model for prognosis prediction. A total of 16 prognosis-associated lncRNAs were screened by LASSO regression analysis (**Figure 4A**) and partial likelihood deviance (**Figure 4B**). Subsequently, we performed multivariate Cox regression analysis on the 16 prognosis-associated lncRNAs based on the AIC value, and finally obtained 7 lncRNAs, namely NR2F1-AS1, ALMS1-IT1, NNT-AS1, LINC00628, SNHG22, STAM-AS1, and CASC2, including 4 high-risk lncRNAs (NR2F1-AS1, ALMS1-IT1, NNT-AS1, and SNHG22) and 3 low-risk lncRNAs (LINC00628, STAM-AS1, and CASC2) (**Figure 4C**). The risk score of each patient in the training set was calculated based on the regression coefficient and expression level of m^6^A- and m^5^C-related prognostic lncRNAs. The expression levels of the eight genes used to construct the risk score in the high and low risk groups are shown in **Figure S2**. Patients with a score lower than the median risk score were categorized into the low-risk group (n = 436), whereas those with a score greater than median risk score were allocated to the high-risk group (n = 436). Compared with subtype B, subtype A showed significantly increased risk score. The distribution of patients in the two subtypes and two risk subgroups is shown in **Figures 4D, E**. The risk distribution plot based on the signature revealed that survival times decreased, whereas mortality rates increased with increasing risk scores (**Figures 4F, G**). Survival analysis revealed that the prognosis of patients in the low-risk score group was better than that in the high-risk score group (log-rank test, p-value < 0.001; **Figure 4H**). In addition, the time-dependent ROC curves showed that the AUC in the training set was 0.721 (**Figure 4I**).

**Figure 5:**
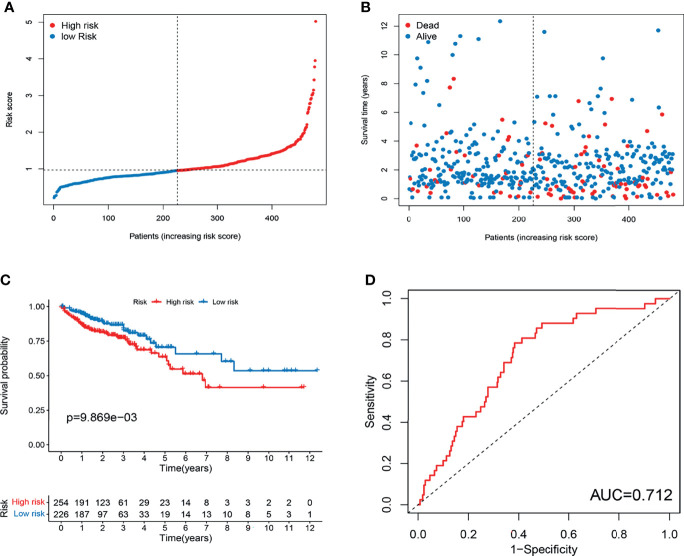
Evaluation of m^6^A- and m^5^C-related lncRNA signature in TCGA set. **(A)** The ranked dot plot indicates the risk score distribution. **(B)** Scatter plot presenting the patient survival status. **(C)** KM analysis of overall survival (OS) between the two groups. **(D)** Receiver operating characteristic (ROC) curve of the m^6^A- and m^5^C-related lncRNA signature.

To validate the prognostic performance of the signature, we calculated risk scores across the TCGA set (**Figure 5A**). Patients were also stratified into high- or low-risk groups based on the same formula as that for the training set. The risk scores and survival status of patients of the low- and high-risk groups are shown in **Figures 5A, B**. Survival analysis revealed a significantly better prognosis in the low-risk group, compared to the high-risk group (log-rank; p-value < 0.05; **Figure 5C**), with the AUC of the ROC greater than 0.7 (**Figure 5D**), indicating that the signature had excellent ability to predict the survival of CRC patients.

### Validation of the Expression Levels of Eight lncRNAs of Prognostic Signature

To further verify the accuracy of the m^6^A- and m^5^C-related lncRNA signature, the expression levels of seven prognostic lncRNAs were measured in six CRC tissues and adjacent normal tissues using RT-qPCR. As shown in **Figure S3**, the expression levels of ALMS1-IT1, NNT-AS1, SNHG22, and STAM-AS1 were significantly upregulated in CRC tissues, whereas NR2F1-AS1, LINC00628, and CASC2 expression was downregulated in CRC tissues compared with that in the corresponding normal tissues.

### Clinical Correlation Analysis and Stratification Analysis of the Prognostic Signature

**Figure 6:**
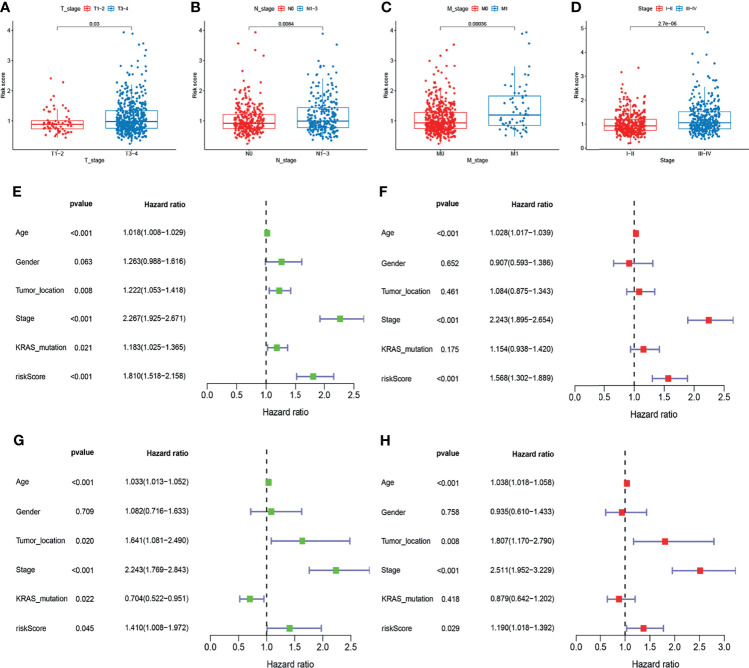
Correlation and independent prognosis analysis of risk score and clinicopathological variables in colorectal cancer (CRC). **(A–D)** Correlation between the risk score and T, N, and M stage, and TNM stage. **(E, F)** Univariate and multivariate analyses showed the prognostic value of the lncRNA signature in the GEO set. **(G, H)** Univariate and multivariate analyses showed the prognostic value of the lncRNA signature in the TCGA set.

To examine the association of the signature with the clinicopathological characteristics of CRC, we determined the correlation between the signature and different clinical characteristics of CRC (age, sex, tumor location, TNM stage, KRAS mutation, and BRAF mutation). The risk scores in the T3-4, N1-3, M1, and stage III-IV, subgroups were significantly higher than those in the stage T0-2, N0, M0, and stage I-II subgroups (p-value < 0.05; **Figures 6A–D**). To determine whether this prognostic signature might independently predict the prognosis for CRC patients, we combined the clinical features (age, gender, tumor location, TNM stage, and KRAS mutation) with the risk scores of the prognostic signature in univariate and multivariate Cox regression analyses. As shown in **Figures 6E. F**, the age, TNM stage, and risk score in the GEO group showed significant differences, and the results were consistent with those in the TCGA group (**Figures 6G, H**). Moreover, to assess whether the signature retains its predictive ability in various subgroups, we stratified subgroups by age (age ≤ 60 and age > 60), gender (female and male), tumor location (left-side and right side), TNM stage (stage I-II and stage III-IV), and KRAS mutation (yes and no). As shown in **Figure S4**, the OS of low-risk patients based on age (p-value < 0.001), gender (p-value = 0.003 in male), tumor location (p-value = 0.008 in left-side and p-value = 0.040 in right side), TNM stage (p-value = 0.045 in stage I-II and p-value < 0.001 in stage III-IV), and KRAS mutation (p-value = 0.022 in yes and p-value = 0.028 in no) was significantly higher than that of high-risk patients.

### m^6^A- and m^5^C-Related lncRNA Signature in Anti-PD-1/L1 Immunotherapy

To investigate whether the signature can predict patient response to immune checkpoint blockade therapy, we calculated the risk score in an anti-PD-L1 cohort (IMvigor210). Patients with a low-risk score exhibited significantly clinical benefits and significantly favorable OS (**Figure S5A**). Furthermore, patients in the CR/PR group had a lower risk score, suggesting that patients in the low risk-score group a significant therapeutic advantages and clinical response to anti-PD-1/L1 immunotherapy compared to those in the high risk-score group (**Figure S5B**).

### Relationship Between the Signature and the CSC Index and Sensitivity to Chemotherapeutic Agents

**Figure 7:**
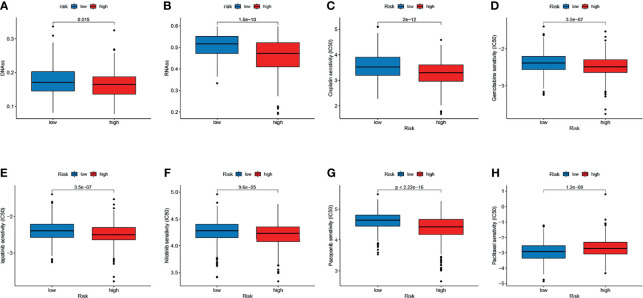
Relationship between the signature and the CSC index and sensitivity to chemotherapeutic agents. **(A, B)** Correlation between the risk score and CSC index. **(C–H)** The IC50 values of six chemotherapeutic drugs in the high- and low-risk groups.

We analyzed the correlation between the two subtypes and the CSC index. As shown in **Figures 7A, B**, there was a significant difference in CSC indexes between high-risk and low-risk groups. Distinct CRC subgroups in the signature should guide clinical treatment. Thus, we compared the sensitivity of high-risk and low-risk groups to common anticancer drugs to identify potential CRC treatment modalities. Patients in the high-risk group may be sensitive to Cisplatin, Gemcitabine, lapatinib, Nilotinib, and Pazopanib, while those in the low-risk group was more sensitive to Paclitaxel (**Figures 7C–H**). Under these circumstances, it is possible that these drugs could be employed for the treatment of CRC with a high risk in the future.

### Development of a Nomogram for Predicting Survival

**Figure 8:**
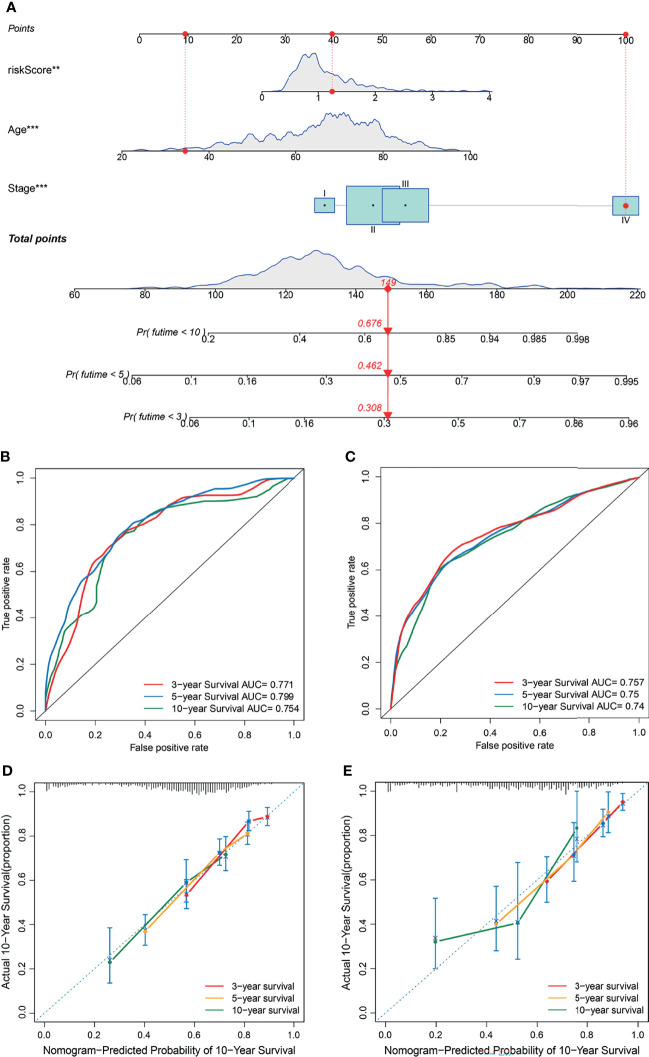
Construction and validation of a nomogram. **(A)** A nomogram for predicting the overall survival (OS) of colorectal cancer (CRC) patients at 3-, 5-, and 10-years in the GEO set. **(B, C)** Receiver operating characteristic (ROC) curves for predicting the 3-, 5-, and 10-year ROC curves in the GEO cohort **(B)** and TCGA set **(C)**. **(D, E)** Calibration curves of the nomogram for predicting 3- and 5-year OS in the GEO cohort **(D)** and TCGA set **(E)**.

To establish a quantitative method to predict the prognosis of CRC patients, we built a nomogram to predict the 3-, 5-, and 10-year OS of CRC patients. All variables that were significant (age, stage, and risk score) in the multivariate analysis were enumerated in the nomogram according to the algorithm. The nomogram displays an example of a patient to predict survival probability. The variable scores were summed to obtain the total points, and the total point line is shown at the bottom of the nomogram, which can predict the probability of OS at 3-, 5-, and 10 years (**Figure 8A**). We performed AUC experiments on the nomogram model and found that it had a higher accuracy in predicting OS at 3-, 5-, and 10-years in the GEO and TCGA sets (**Figures 8B, C**). Calibration curves were drawn to depict the predictive value between the predicted 3-, 5-, and 10-year survival events and the virtual observed outcomes in the GEO and TCGA sets (**Figures 8D, E**), which showed that the nomogram model was highly accurate, affirming its practicability in predicting patient prognosis.

### GO and KEGG Analysis

To determine the potential biological processes and signaling pathways related to the signature, we performed GO terms and KEGG pathway analysis of the low- and high-risk groups. The “limma” R package was used to identify the differentially expressed genes (DEGs) between the high- and low-risk groups with the criteria of |logFC| > 1 and adjusted p-value < 0.05. The results of GO functional annotation analysis of the DEGs showed that the most significantly enriched biological processes included extracellular matrix organization, extracellular structure organization, and external encapsulating structure organization. The most significantly enriched cellular components included collagen-containing extracellular matrix, endoplasmic reticulum lumen, and apical part of cell. The most significantly enriched molecular functions included extracellular matrix structural constituent, glycosaminoglycan binding, and sulfur compound binding (**Figure S6A**). KEGG pathway enrichment analysis for the DEGs showed that the significantly enriched pathways included PI3K-Akt signaling pathway, the ECM-receptor interaction, focal adhesion, proteoglycans in cancer, and Transcriptional misregulation in cancer (**Figure S6B**; **Table S6**). Notably, most of these functions were significantly correlated with the occurrence and development of tumors.

## Discussion

CRC is a heterogeneous and highly malignant tumor with high morbidity and mortality (37). Owing to the phenotype and genetic heterogeneity of CRC, the accuracy of conventional methods using clinical features in predicting individual outcome and survival is still limited. Accurate prognostic prediction and individualized clinical treatment strategy are the basis of precision medicine (38). Most of the established clinical markers for treatment response and prognosis of CRC are based on clinical features, and their accuracy and specificity are limited. Traditional AJCC TNM staging is mainly based on anatomical information and cannot adequately assess the prognosis of CRC patients. Therefore, exploring the molecular mechanisms and screening reliable CRC-specific genomic signatures are urgently needed to improve prognosis assessment and individualized treatment.

Following in-depth studies of post-transcriptional modifications, researchers gradually realized the importance of epitranscriptomics in CRC. Increasing studies have confirmed and highlighted the potential effects of several common modifications in lncRNAs (m^6^A and m^5^C) on cancer development and progression. As the most abundant post-transcriptional modification in eukaryotic ncRNAs, m^6^A has a huge effect on their maturation, export, stability, translation, export, and decay (13, 14). Previous studies have shown that m^6^A “writers” and “erasers” could adjust the levels of m^6^A modification in lncRNAs to regulate binding sites to m^6^A “reader” proteins. Different m^6^A “reader” proteins recognize and bind to methylated lncRNAs to exert different functions. Liu et al. (39) revealed that the specific m^6^A readers YTHDF1 and YTHDF2 can read m^6^A motifs and regulate the stability (and decay) of the lncRNA THOR, thereby regulating the proliferation, migration, and invasion of cancer cells. The m^6^A regulators reportedly act as a lncRNA structural switch, participate in the lncRNA-mediated competing endogenous RNA model, and enhance the stability of lncRNA to serve its functions, thereby influencing tumor initiation and progression. For example, m^6^A-induced LNCAROD can promote the development of head and neck squamous cell cancer by forming a ternary complex with YBX1 and HSPA1A (40). The m^6^A mark increased the stability of lncRNA FAM225A, which promotes nasopharyngeal carcinoma progression by acting as ceRNA to sponge miR-590-3p/miR-1275 (41).

m^5^C is distributed widely in lncRNAs and involved in various biological processes related to the occurrence and progression of tumors. In a previous study, quantitative mapping of m^5^C sites in *Arabidopsis thaliana* on a transcriptome range revealed more than 1000 m^5^C sites in mRNA, lncRNAs, and other non-coding RNAs (42). He et al. (43) found that m^5^C methylation in lncRNAs occurred more frequently in hepatocellular carcinoma than in the adjacent non-tumor tissues, and a higher number of methylated genes were upregulated. Sun et al. (44) revealed that H19 lncRNA modified by the m5A “writer” NSUN2 promoted the occurrence and development of hepatocellular carcinoma by recruiting the G3BP1 oncoprotein. These lncRNAs can be upregulated or downregulated to promote cancer cell proliferation and migration. Although significant progress has been made in the field of epitranscriptomics and lncRNA research, little is known about the functional role of lncRNAs in cancers or their complete mechanism of action. To advance individualized therapies based on lncRNAs, it is important to clarify the interactions between the chemical modifications that occur in lncRNAs. Moreover, it is necessary to further study m^6^A- and m^5^C-related lncRNAs to clarify their potential regulatory mechanism in the TME.

In the present study, we identified 1401 m^6^A- and m^5^C-related lncRNAs by Pearson’s correlation analysis between 23 m^6^A regulators, 15 m^5^C regulators, and lncRNAs, and we screened out 21 prognostic lncRNAs. We detected two distinct molecular subtypes based on the 21 prognostic lncRNAs, and determined that cluster A was significantly associated with advanced clinicopathological features and worse survival outcomes. The TME characteristics and the proportions of 22 TIICs were significantly different between the two subtypes. This CRC subtype was also characterized by a significant stroma activation status, including the EMT and TGF-β signaling pathways. These findings suggested that m^6^A- and m^5^C-related lncRNAs might serve as a valid prognostic biomarker and predictor for evaluating the clinical outcome and immunotherapy response of CRC patients. Therefore, we conducted LASSO Cox regression and multivariate Cox proportional hazard regression analyses to construct a robust and effective prognostic signature. Patients in the high- and low-risk groups divided by risk scores exhibited significantly different clinicopathological characteristics and survival outcomes. In addition, there was a significant difference in CSC indexes between high-risk and low-risk groups. We also found that patients in the high-risk group may be sensitive to Cisplatin, Gemcitabine, lapatinib, Nilotinib, and Pazopanib. Under these circumstances, it is possible that these drugs could be employed for the treatment of CRC with a high risk in the future. Functional enrichment analysis showed that these lncRNAs related to the high-risk group were involved in the development and progression of CRC. Last, a nomogram was constructed based on age, tumor stage, and risk score to further improve the performance and facilitate the use of the m^6^A- and m^5^C-related lncRNA signature. The m^6^A- and m^5^C-related lncRNA signature can be used for prognosis stratification of CRC patients, and will assist with understanding the molecular mechanism of CRC and provide new ideas for targeting therapies.

The immune system plays a complex role in cancer development and substantially affects CRC progression. The prognosis of CRC after conventional chemotherapy is poor, with high levels of tumor neoantigens, tumor infiltrating lymphocytes, and checkpoints. There is growing evidence that the TME, in which immune cells and molecules are important components, plays a crucial role in tumor development, and that the degree of immune cell infiltration is highly correlated with patient prognosis (45). The TME that surrounds tumor cells is composed of TIICs, mesenchymal cells, endothelial cells, inflammatory mediators, and ECM molecules (46). Evidence has shown that the TME has significant effects on tumor growth and development, therapeutic resistance, and clinical outcome (47). In this study, we discovered that the TME characteristics and proportions of 22 TIICs were significantly different between the two molecular subtypes. This suggests the critical role of m^6^A- and m^5^C-related lncRNAs in CRC progression. A previous study showed that high density of plasma cells can predict a relatively auspicious signal for the prognosis of CRC patients (48), which is consistent with our finding that plasma cells were more clustered in subtype B than in subtype A. Previous studies revealed that tumor-infiltrating B cells were associated with favorable outcomes in CRC (48, 49). Patients with metastatic CRC who exhibit high infiltration of B cells have significantly lower risk for disease recurrence and prolonged OS (49). In our study, there was no significant difference in the degree of naive B cell infiltration between the two subtypes, but the infiltration level of memory B cells in subtype A, which had worse OS, was significantly lower than that in subtype B. This indicates that B cell infiltration inhibits tumor invasion and metastasis in CRC, consistent with previous studies (48, 49).

Increasing evidence shows that memory T cells, effector T cells, and T cell differentiation play an important role in the immune defense of CRC (50). T cells can be classified into CD4+ and CD8+ T cells, and the former can further differentiate into regulatory T cells (Tregs) and follicular helper T cells. Tregs are responsible for maintaining the balance of immune responses and preventing excessive immune responses, and they are thought to be involved in the escape of cancer from the host immune system [51]. Gamma delta T cells can effectively recognize and kill CRC cells, thereby suppressing tumor progression via multiple mechanisms (51). The densities of tumor-infiltrating T cells in CRC tissues were higher than that in the normal tissues, and higher densities indicated a good prognosis (51–53). In this study, subtype B, which had a better prognosis, exhibited higher immune infiltration of CD4+ and CD8+ T cells, suggesting that they play a positive role during CRC development. Tumor-associated macrophages are divided into two main phenotypes: M1 macrophages (which inhibit cancer progression) and M2 macrophages (which promote cancer progression). M1 macrophages participate in a positive immune response and exert the function of immune surveillance by secreting proinflammatory cytokines and chemokines as well as presenting antigens. M2 macrophages have weak antigen-presenting ability and participate in immune regulation by secreting inhibitory cytokines to downregulate the immune response (54). CRC has a high level of MMP-9, which can degrade collagen in the type IV basement membrane, thereby promoting metastasis (55). In this study, we found that infiltration of M1 macrophages was higher in subtype B with a better prognosis, whereas the level of M2 macrophage infiltration was higher in subtype A with a poorer prognosis, consistent with known findings.

In recent years, with in-depth research on tumor immunology and molecular biology, immunotherapy has provided a new direction for tumor treatment. Tumor immunotherapy aims to activate the human immune system, kill tumor cells and tissues through autoimmune function, and restore the normal antitumor immune response of the body by restarting and maintaining the tumor-immune cycle to control and eliminate tumors (56, 57). This therapy includes monoclonal antibody immune checkpoint inhibitors, therapeutic antibodies, cancer vaccines, cell therapy, and small-molecule inhibitors. Recently, immune checkpoint therapies targeting PD-1 (PDCD1), PD-L1, CTLA-4, and HAVCR2 have gained attention, and clinical studies have shown that they are reliable in terms of safety and efficacy (58, 59). Mismatch repair-defective CRC account for 14% of all CRCs (60). Patients with microsatellite instability have a higher response to PD-1 treatment. In this study, we found that the proportion of microsatellite instability was higher in subtype B with a poor prognosis. Immune checkpoint inhibitors have recently been used to treat CRC (61). In this study, we also conducted correlational analysis for the two subtypes and the expression of tumor immune checkpoint genes (PD-1, PD-L1, CTLA-4, and HAVCR2), and our results revealed that subtype A was positively associated with the expression of PD-L1, HAVCR2, and CTLA-4. PD-1 is an inhibitory receptor expressed on the surface of activated T cells with two ligands, PD-L1 and PD-L2. PD-1 acts as a “brake” in tumor immunity and inflammation reactions. PD-L1 is generally widely expressed on the surface of epithelial cells, endothelial cells, and tumor cells. In this study, we found that patients with low risk scores exhibited significant therapeutic advantages and clinical response to anti-PD-1/L1 immunotherapy compared to those with high-risk scores, suggesting that the established signature will contribute to predicting patient responses to anti-PD-1/L1 immunotherapy. CTLA-4 exists on the surface of T cells and can prevent B7 from binding to the CD28 receptor on T cells, thereby inhibiting immune stimulation (62). PD-1, PD-L1, and CTLA-4 inhibitors have been widely applied for different types of solid tumors (63–65). Tremelimumab is an anti-CTLA-4 monoclonal human antibody that is well-tolerated by CRC patients who have poor response to other immunotherapies (66). HAVCR2, namely, TIM-3, which inhibits tumor immunity with depletion of T cells, is a negative regulator of immune check points. The immune checkpoint blockade for HAVCR2 has achieved encouraging efficacy in the medical treatment of advanced non-small cell lung cancer (67) and hepatocellular carcinoma (68). Thus, we can conclude that the high-risk CRC patients with higher expression of PD-L1, CTLA-4, and HAVCR2 may respond to immune checkpoint blockade.

The proposed signature contained eight m^6^A- and m^5^C-related lncRNAs. Among the seven lncRNAs, four have been shown to be involved in the development and progression of in CRC and other kinds of malignancies, namely NNT-AS1, CASC2, ALMS1-IT1, SNHG22, LINC00628, and NR2F1-AS1. NNT-AS1 is an oncogene associated with worse OS in CRC. Wang et al. (69) revealed that NNT-AS1 was significantly upregulated in CRC tissues and was clearly linked to clinical stage, lymph node metastasis, vessel invasion, and worse OS and progression-free survival. Silencing of NNT-AS1 suppressed cell proliferation, migration, and invasion by activating the MAPK/Erk and EMT signaling pathways. Moreover, a high NNT-AS1 expression level was also observed in the serum and exosomes of CRC patients, and was associated with an advanced tumor stage. Knockdown of NNT-AS1 impaired the proliferation, migration, and invasion of CRC cells *via* regulation of the miRNA-496/Ras-related protein Rap-2c axis (70). In this study, NNT-AS1 was found to be a risk factor, which is inconsistent with the findings of previous studies. LncRNA CASC2 has been reported to be a tumor suppressor in CRC, and its low expression was significantly more frequent in the advanced TNM stage. Its overexpression suppressed proliferation of CRC cell and tumor growth *via* the miR-18a/STAT3 axis (71). CASC2 was also found to enhance berberine-induced cytotoxicity in CRC cells by silencing BCL2 (72). Luan and colleagues recently reported that ALMS1-IT1 promoted the malignant progression of lung adenocarcinoma through AVL9-mediated activation of the cyclin-dependent kinase pathway (73). SNHG22 has been detected to be overexpressed and to act as an oncogene in multiple cancers, including CRC (74, 75). Yao et al. (75) revealed that SNHG22 promoted CRC cell growth, migration, and invasion through SNHG22/miR-128-3p/E2F3 axis. LINC00628 has been reported to inhibit the malignant progression of cancer through different mechanisms, such as binding to EZH2 to regulate the p57 or H3K27me3 level (76, 77), and interacting with the promoter of LAMA3 or VEGFA (78, 79). In present study, we also found that LINC00628 was CRC suppressor, which is inconsistent with the findings of previous studies.

## Conclusions

Our comprehensive analysis of two types of RNA modifications revealed their potential functions in the tumor-immune-stromal microenvironment, cancer clinicopathological features, and cancer prognosis, and we determined their therapeutic liability in targeted therapy and immunotherapy. These findings highlight the crucial clinical implications of the cross-talk of m^6^A- and m^5^C-related lncRNAs and provide new ideas for guiding personalized immune immunotherapy strategies for CRC patients.

## Data Availability Statement

The datasets analyzed for this study can be found in the TCGA-COAD/READ project (http://www.cancer.gov/tcga) and GEO (https://www.ncbi.nlm.nih.gov/geo/query/acc.cgi?acc=GSE39582/GSE17536/GSE38832).

## Author Contributions

WS, JR, RSX, and TF made substantial contributions to the conception, design, interpretation, and preparation of the final manuscript. WS, JR, RSX, and TF participated in the coordination of data acquisition and data analysis, and reviewed the manuscript.

## Funding

This work was supported by the Talent Introduction Fund of Wuhan University Renmin Hospital (grant number NA to TF).

## Conflict of Interest

The authors declare that the research was conducted in the absence of any commercial or financial relationships that could be construed as a potential conflict of interest.

## Publisher’s Note

All claims expressed in this article are solely those of the authors and do not necessarily represent those of their affiliated organizations, or those of the publisher, the editors and the reviewers. Any product that may be evaluated in this article, or claim that may be made by its manufacturer, is not guaranteed or endorsed by the publisher.
