# Supplementary material for: Cross-Talk Between m6A- and m5C-Related lncRNAs to Construct a Novel Signature and Predict the Immune Landscape of Colorectal Cancer Patients
Source: Front Immunol. 2022 Mar 8;13:740960. doi: 10.3389/fimmu.2022.740960 (PMC8957790; doi:10.3389/fimmu.2022.740960)
Supplement: Supplementary file 1 [file Image_1.pdf]

Supplementary Figures

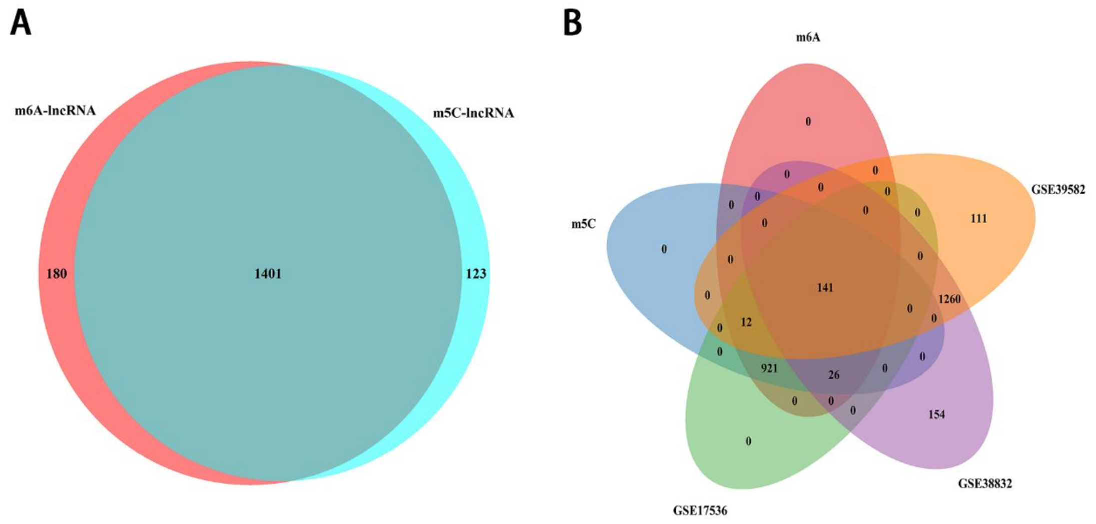

Figure S1 Identification of m6A- and m5C-related lncRNAs in CRC. (A) The intersection of 1524 m6A- and 1581 m5C-related lncRNAs. (B) 141 common lncRNAs of m6A- and m5C-related lncRNAs of four datasets (TCGA-COAD/READ, GSE39582, GSE17536, and GSE38832).

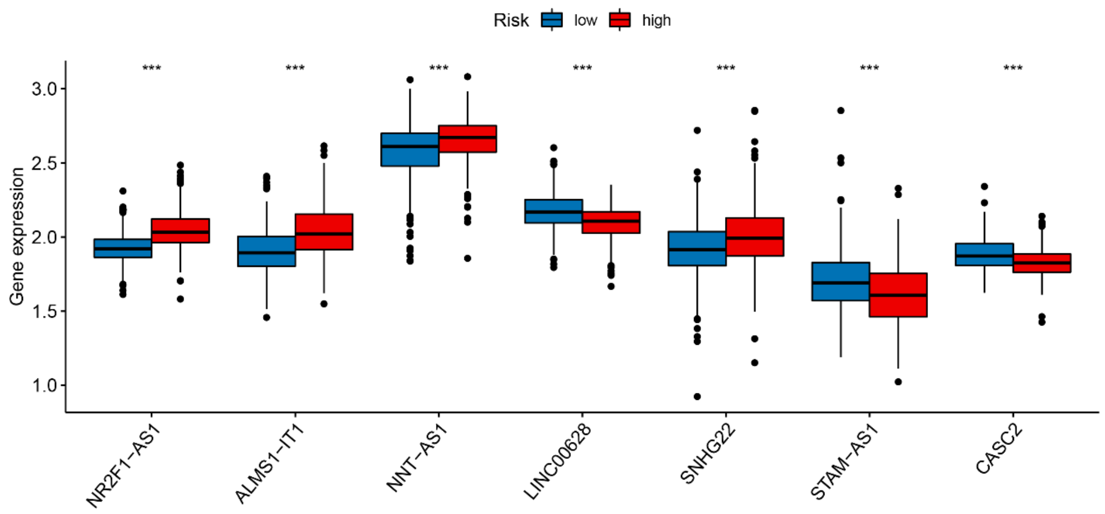

Figure S2 The expression levels of the seven genes used to construct the risk score in the high and low risk groups.

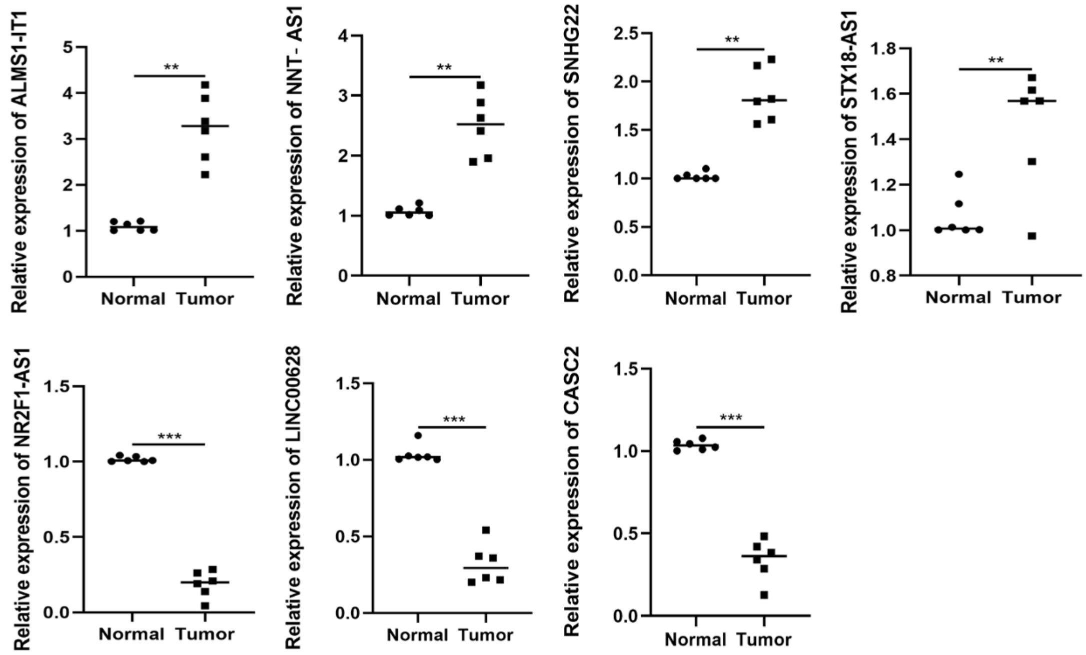

Figure S3 Expression levels of 7 m6A- and m5C-related lncRNA signature in colorectal cancer tissues and corresponding normal tissues by RT-PCR.

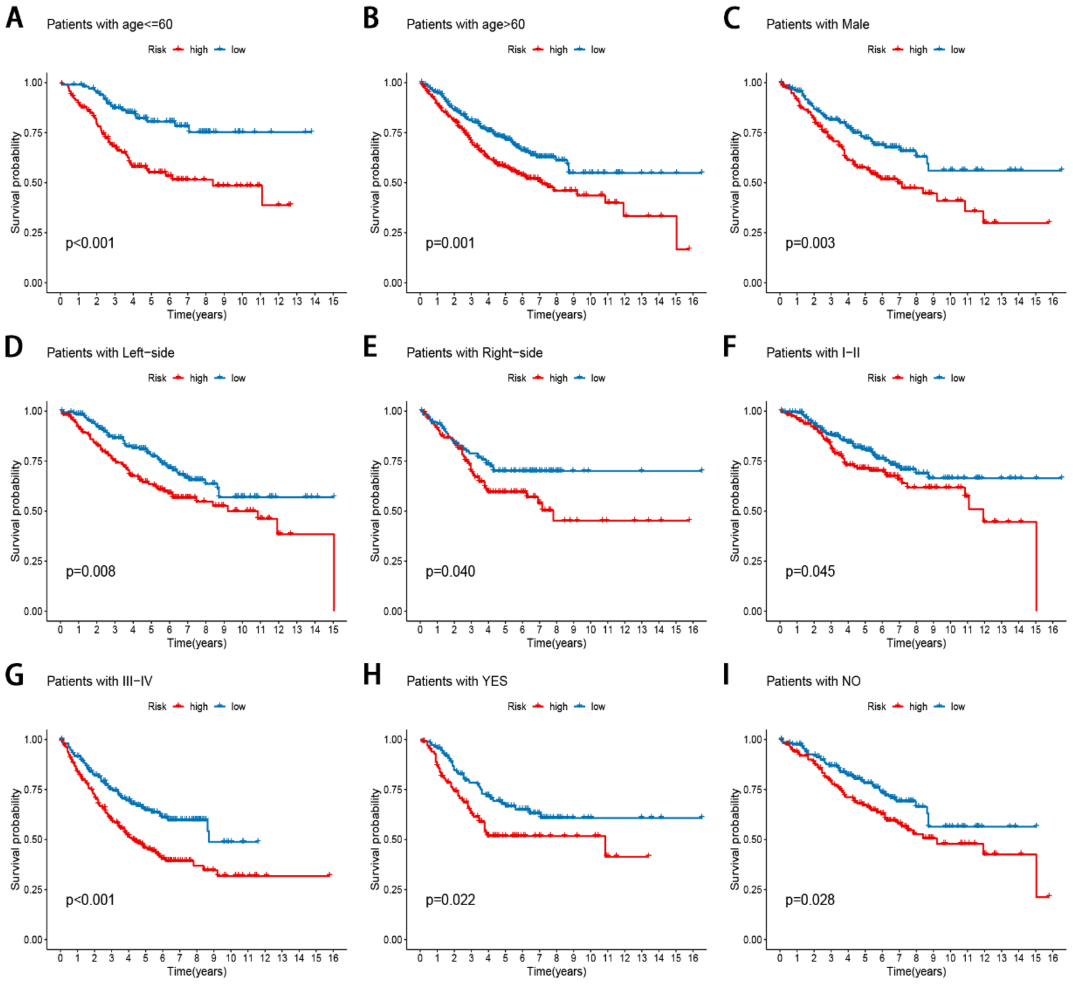

Figure S4 Stratification analysis of the m6A- and m5C-related lncRNA signature in CRC. (A-B) Age (age ≤ 60 and age > 60 years old). (C) Gender (male). (D-E) Tumor location (left-side or right-side). (F-G) Tumor stage (I-II or III-IV). (H-I) KRAS mutation (yes and no).

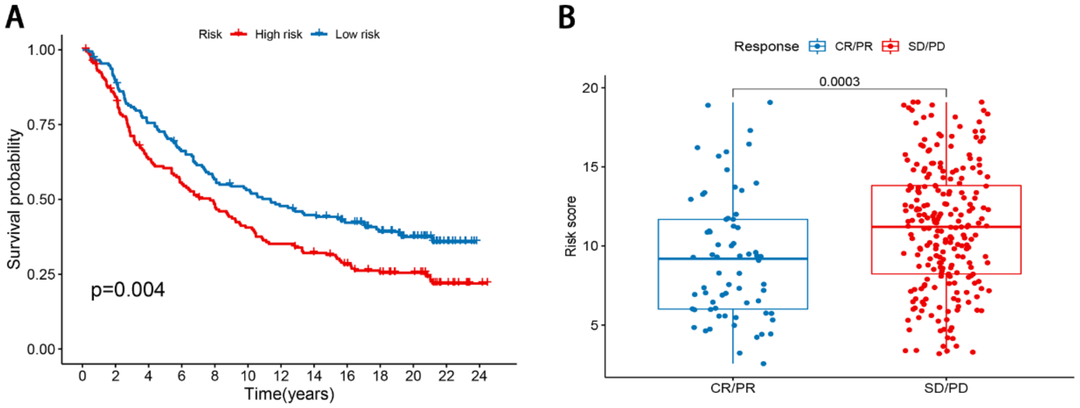

Figure S5 m6A- and m5C-related lncRNA signature in the role of anti-PD-1/L1 immunotherapy. (A) Survival analyses for low and high risk score patient groups in the anti-PD-L1 immunotherapy cohort using Kaplan-Meier curves. (B) Distribution of risk score in distinct anti-PD-L1 clinical response groups. SD, stable disease; PD, progressive disease; CR, complete response; PR, partial response.

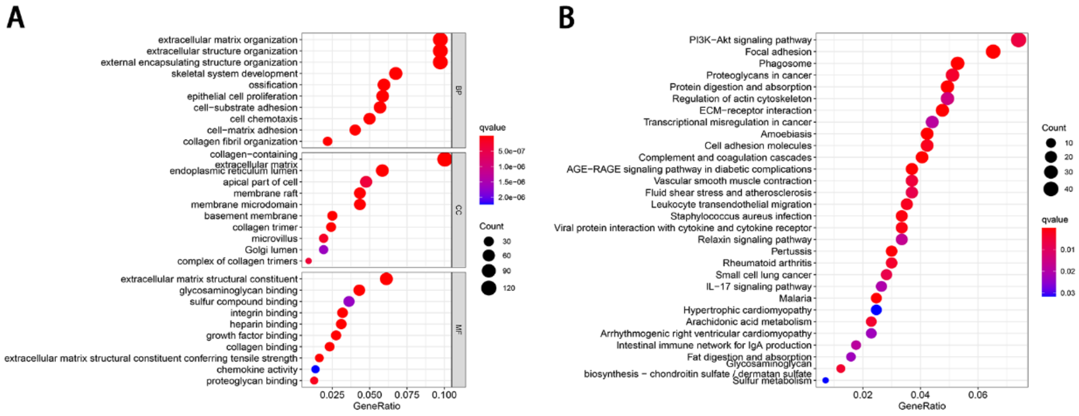

Figure S6 Functional annotation of the differentially expressed genes between the two risk groups. (A) Top 10 GO enrichment of the differentially expressed genes. (B) Top 30 enriched KEGG pathways of the differentially expressed genes.
